# Healthcare providers’ knowledge, attitudes and practices on smoking cessation intervention in the Northern Cape

**DOI:** 10.4102/hsag.v29i0.2489

**Published:** 2024-01-24

**Authors:** Lizwe C. Muza, Chika K. Egenasi, Wilhelm J. Steinberg, Mathew O. Benedict, Talat Habib, Ferdinand Mampuya, Cornel van Rooyen

**Affiliations:** 1 Department of Family Medicine, Faculty of Health Sciences, University of the Free State, Bloemfontein, South Africa; 2 Department of Family Medicine, Robert Mangaliso Sobukwe Hospital, Kimberley, South Africa; 3 Department of Biostatistics, Faculty of Health Sciences, University of the Free State, Bloemfontein, South Africa

**Keywords:** smoking, knowledge, attitude, practice, Sol Plaatje’s, Northern Cape, barrier, clinic

## Abstract

**Background:**

Clinicians are crucial in encouraging smokers to quit through behavioural or pharmacological smoking cessation interventions. Smokers quit better with professional help. The level of healthcare providers’ (HCPs) knowledge, attitudes and counselling skills related to effective smoking cessation support in the study area remains uncertain.

**Aim:**

The study aimed to determine HCPs’ knowledge, attitudes and practices (KAP) on smoking cessation intervention strategies in the Sol Plaatje district, Northern Cape.

**Setting:**

Sol Plaatje’s 13 district municipality clinics, Kimberley, Northern Cape.

**Method:**

A descriptive, cross-sectional analytical study involving healthcare workers in the above setting.

**Results:**

A total of 165 HCPs, including medical officers, professional nurses, enrolled nurses and assistant-enrolled nurses working in primary healthcare clinics, were invited to participate in the study, with 156 completing the questionnaires. About 53.9% had no knowledge of South African tobacco smoking cessation guidelines, while 87.2% knew the importance of counselling patients about smoking and its impact on quitting. The majority of them did not know the medicines recommended for tobacco treatment in South Africa. About 89.7% expressed that smoking cessation counselling is an important part of their jobs. However, less than half indicated that they make follow-up arrangements for those attempting to quit.

**Conclusion:**

The study revealed gaps in KAP regarding smoking cessation among the respondents, necessitating the need for continuing education on the existing smoking cessation guidelines among these HCPs.

**Contribution:**

The results of this study will help to improve smoking cessation intervention knowledge among health providers.

## Introduction

Tobacco use remains a significant public health risk worldwide and is associated with a wide range of medical conditions, which are preventable yet still claim over eight million lives annually (National Institute on Drug Abuse 2022). Stopping smoking is not easy because the nicotine in smoke is highly addictive (U.S. Department of Health and Human Services 2020). The benefits of quitting, however, are almost immediate, with a lowering of blood pressure and heart rate, improved taste and smell, and a longer-term reduction in risk of cancer, heart attack and chronic obstructive pulmonary disease (COPD) (American Cancer Society 2020; U.S. Department of Health and Human Services 2020). Equipped with adequate knowledge, the right attitude and practices, healthcare providers (HCPs) can facilitate smoking cessation by assisting those who want to quit, a role strongly recommended by the World Health Organization’s Framework Convention on Tobacco Control (WHO FCTC 2021).

The World Health Organization (WHO) has made significant progress in the fight against tobacco use through its six ‘MPOWER’ measures, which include monitoring tobacco use and preventive measures, protecting people from tobacco smoke, offering help to quit, warning about the dangers of tobacco, enforcing bans on advertising, promotion and sponsorship and raising taxes on tobacco (Zhang et al. 2022). The WHO recommends governments to implement regulations to stop non-smokers from starting to use electronic nicotine delivery systems (ENDS), such as ‘e-cigarettes’, to prevent the renormalisation of smoking in the community and to protect future generations (World Health Organization 2021).

Countries in sub-Saharan Africa are experiencing an increasing rate of tobacco use because of factors such as fast population growth, increased consumer purchasing power and intensive efforts by the tobacco industry to expand African markets (Egbe et al. 2022). The primary tobacco control law in South Africa is the ‘Tobacco Products Control Act 83 of 1993’, with several subsequent amendments (South African Government 1993). This act intends to prohibit or restrict smoking in public places, regulate the sale and advertising of tobacco products in certain respects and prescribe what should be reflected on packages (South African Government 1993). South Africa also became a signatory to the ‘WHO FCTC’ on 18 July 2005 (Tobacco Control Laws 2021). The Global Adult Tobacco Survey (GATS-SA) conducted by the South African Medical Research Council showed that 25.8% of South Africans were smokers in 2021, a drop from 32% in 1993 (SAMRC 2022). However, tobacco-related illnesses are estimated to cause over 31 000 deaths and cost the economy approximately R42 billion annually (Nojilana et al. 2022). In December 2022, the ‘Tobacco Products and Electronic Delivery Systems Control Bill’ was introduced before parliament, aiming to enforce stricter controls on tobacco products and ENDS in future (Parliament of South Africa 2022). However, much work remains to assist those who wish to quit smoking.

Healthcare providers can be crucial in smoking cessation interventions in all settings (Pipe, Evans & Papadakis 2022). They can assist patients in quitting by providing advice, brief counselling, prescribing cessation medications and referring them to additional resources, such as a helpline (Devonish et al. 2022). Additionally, they can provide continued support to prevent relapse (Devonish et al. 2022). Primary care physicians, dentists, nurses and other health professionals have been involved in smoking cessation interventions in the United States, Europe, Australia, South Africa and other countries (Ayo-Yusuf & Omole 2021; Gajendra, McIntosh & Ghosh 2023; Mersha et al. 2022). Several evidence-based guidelines are available to assist health professionals with tobacco cessation interventions (Verbiest et al. 2017).

A review of 26 national guidelines in primary care for smoking cessation from 22 countries by Verbiest et al. (2017) revealed almost universal agreement regarding the importance of identifying smokers, offering advice and providing behavioural and pharmacological support to quit. The South African National Smoking Cessation Guidelines, for instance, include the following key steps: identifying all smokers, alerting them to the harms of smoking and the benefits of quitting, assessing readiness to initiate an attempt to quit, assessing the physical and psychological dependence to nicotine and smoking, determining the best combination of counselling, support and pharmacological therapy (Van Zyl-Smit et al. 2013). South African HCPs can access various pharmacological intervention strategies such as nicotine replacement therapy (NRT), antidepressants, nicotine receptor agonists and complementary medicines, for example, hypnotherapy and acupuncture (Van Zyl-Smit et al. 2013). A person who tries to quit smoking through the assistance of HCPs has more chance of quitting smoking than those who use NRT alone or any other means (Van Zyl-Smit et al. 2013).

Current literature suggests that despite evidence-based guidelines and recommendations being available nationally and internationally, there is still a gap between evidence and practice when assessing tobacco use and supporting patients to quit (Ayo-Yusuf & Omole 2021; Jradi 2017; Tadzimirwa et al. 2019). In the GATS-SA survey in 2021, 65.7% of current South African smokers expressed an interest in quitting, but only 42.9% of smokers who visited their physician in the past 12 months received cessation advice (SAMRC 2022). Several local and international studies have identified common barriers to implementing evidence-based recommendations for tobacco cessation interventions, such as lack of HCP knowledge and skills, time constraints, clinician attitudes, beliefs and practice norms, and a lack of incentives, among others (Jradi 2017; Pipe et al. 2022).

To provide sound advice on smoking cessation, HCPs must be knowledgeable and possess the right attitude, which is crucial to giving meaningful advice. In a recent systematic review, Tildy et al. (2023) found that a lack of knowledge among health providers and inadequate messaging about cessation support options are significant barriers to successful smoking cessation interventions (Tildy et al. 2023). This finding assesses HCPs’ knowledge, attitudes and practices (KAP) on smoking cessation interventions. Studies published in the United States, France, Australia, Qatar, Nigeria and other countries have provided ample evidence that clinicians’ knowledge and attitudes about various aspects of smoking cessation influence the degree of intervention offered by a particular provider (Almulla, Kouyoumjian & ElNakib 2021; Bold et al. 2022; Coindard et al. 2022; Mersha et al. 2022; Omotowo, Ndibuagu & Ezeoke 2016). The same conclusion was reached in a study conducted by Omole, Ayo-Yusuf and Ngobale (2014) in South Africa.

To successfully understand the state of the application of the policy on smoking cessation, it was necessary to deduce the level of knowledge, attitude as well as practices of HCPs. While a guideline was developed by Van Zyl-Smit et al. (2013) to guide smoking cessation policy in the country, no research was conducted to check the knowledge of the guidelines by public HCPs in the Northern Cape. This province has the second-highest smoking prevalence rate after the Western Cape (Fagbamigbe et al. 2020). This study aimed to assess the KAP of primary healthcare nurses and doctors on smoking cessation interventions in the Northern Cape, Sol Plaatje district.

## Methods

The study was a descriptive cross-sectional study using a self-administered questionnaire.

### Study population

The Sol Plaatje district is part of the Frances Baard District Municipality of the Northern Cape Province, South Africa. It is named after Solomon Tshekisho Plaatje and includes the diamond mining city of Kimberley (The Local Government Handbook: South Africa 2023). The study population consisted of HCPs (medical officers, professional, enrolled or assistant nurses) working in the 13 Sol Plaatje District Municipality clinics, as obtained from the human resource employment database. The clinics include Galeshewe Community Health Centre, Masakhane Clinic, Beaconsfield Clinic, Betty Gaetsewe Clinic, Florianville Clinic, Mapule Matsepane Clinic, City Clinic, Dr Windston Torres Clinic, Greenpoint Clinic, Ritchie Clinic, Platfontein Clinic, Phutanang and Madoyle Clinic.

### Inclusion and exclusion criteria

Medical officers and professional, enrolled or assistant nurses working in one of the 13 public clinics in Sol Plaatje District Municipality were included in the study. Healthcare providers not working in primary healthcare clinics were excluded.

### Sample size

The study included everyone who satisfied the inclusion criteria; a power calculation to determine an adequate sample size was unnecessary. The intended sample for the study consisted of a maximum of 165 respondents consisting of 18 medical officers, 99 professional nurses, 13 enrolled nurses and 35 enrolled nursing assistants. Only 156 of these HCPs completed the questionnaires from 01 to 28 June 2019 (94.5% response rate).

### Measurements

A confidential, structured, closed-ended questionnaire was used for the research instrument. The questionnaire was designed based on information from prior studies assessing the KAPs of HCPs in smoke cessation intervention (Cohen, McGinnis & Salsberg 2007; Delucchi, Tajima & Guydish 2009; Fiore et al. 2008; Van Zyl-Smit et al. 2013).

The questionnaire consisted of five sub-sections: The ‘*knowledge’* section contains 19 questions designed to evaluate the clinician’s level of knowledge on smoking cessation intervention in the following areas: (1) smoking cessation methods; (2) benefits of smoking cessation interventions; (3) nicotine dependence and withdrawal symptoms; (4) effect of smoking on an unborn child; (5) medications recommended for treatment of tobacco dependence in South Africa. Most questions required a ‘true’, ‘false’ or ‘don’t know’ answer. Knowledge was categorised using Bloom’s cut-off point. This categorisation grades 80% and above as good knowledge, 60% – 79% as moderate and less than 60% as poor knowledge.

The ‘*attitude*’ section consisted of 12 items designed to assess the following: (1) the role of HCP in smoking cessation intervention; (2) the priority between health concerns and smoking cessation intervention; (3) the attitude of smokers to smoking cessation advice; (4) time constraints to provide smoking cessation advise during routine consultations; and (5) smoking status to be included as one of the ‘vital signs’ for patients attending primary care centres. Respondents were required to agree or disagree with the statements.

The ‘*practise’* section was based on the South African tobacco smoking cessation clinical practice guideline, particularly the 5A’s strategy for providing smoking cessation, that is, ask, alert, assess, assist and arrange follow-up. The respondents were asked to state the frequency with which they performed the individual components of the 5A’s strategy in their day-to-day interactions with patients using the scales ‘Always’, ‘Sometimes’ and ‘Never’.

In assessing barriers in smoking cessation interventions, the primary investigator used a series of questions which required the respondents to rate each barrier as either ‘not a barrier’, ‘somewhat a barrier’ or ‘important barrier’.

### Pilot study

The study was piloted at Robert Mangaliso Sobukwe Hospital Kimberley (RMSH). A medical officer, two professional nurses, two nurse assistants and five enrolled nurse assistants completed the questionnaires. However, data obtained from the pilot study were not included as changes were made to the datasheet. They included correction of grammatical errors and spelling out words in full rather than using abbreviations, which was confusing for some of the respondents.

### Data analysis

The Department of Biostatistics, Faculty of Health Sciences, University of the Free State (UFS) assisted with data analysis using Statistical Analysis System (SAS) version 9 (SAS Institute Inc., Cary, North Carolina, U.S.). Frequencies and percentages were calculated for categorical data, and the presentation was performed using tables. Association between variables were assessed using Chi-squared or Fisher’s exact tests. A *p*-value of < 0.05 was set to be the level of significance.

### Ethical considerations

The protocol for this study was approved by the Health Science Research Ethics Committee (HSREC), University of the Free State, with approval number (UFS-HSD2018/1351/2901). Permission for the study was also obtained from the Northern Cape Province ethics committee.

Number coding was used to ensure the confidentiality of the participant’s responses. No names or personal identifiers appeared on any research-related information or datasheet sent for statistical analysis. The researcher kept all paper-based records in a secure location, only accessible to those involved in the study. All information was managed confidentially.

## Results

Of the 165 questionnaires distributed to the maximum number of respondents, 156 were completed by the respondents, giving a response rate of 94.5%. Despite numerous verbal reminders, the remaining nine invited participants (5.5%) did not return the questionnaire.

Table 1 indicates that most respondents were females, within the age brackets 51 years to 60 years, were mostly professional nurses, had over 10 years of work experience, and had never smoked.

**TABLE 1:** Demographic and background characteristics of respondents.

Variables	*n*	%
**Gender**		
Male	29	18.6
Female	127	81.4
**Age category**		
21–30	21	13.5
31–40	36	23.1
41–50	44	28.2
51–60	47	30.1
> 60	8	5.1
**Profession or rank**		
Medical officer	18	11.5
Professional nurse	94	60.3
Enrolled nurse	12	7.7
Enrolled nursing assistant	32	20.5
**Years of practice**		
0–5	28	18.0
6–10	37	24.0
> 10	91	58.0
**Smoking status**		
Never smoked	125	80.1
Ex-smoker	16	10.3
Current smoker	15	9.6

Table 2 shows the respondents’ knowledge scores. The majority of the respondents were not aware of the existence of the South African tobacco smoking cessation clinical practice guidelines.

**TABLE 2:** Respondents’ knowledge score.

Knowledge item		True (%)	False (%)	Don’t know (%)	Yes	No	Don’t know
1.	South Africa has tobacco smoking cessation clinical practice guidelines?	46.1	7.1	46.8	-	-	-
2.	Patients should only be asked about their smoking history if they have a smoking related disease or illness.	17.3	81.4	1.3	-	-	-
3.	Smoking cessation is not a critical component of management of chronic obstructive pulmonary disease.	7.7	85.9	6.4	-	-	-
4.	Smoking cessation advice given by a health professional to a patient increases the patient’s chances of quitting.	87.2	5.1	7.7	-	-	-
5.	It is not necessary to assess nicotine dependence prior to initiation of nicotine replacement therapy	9.6	67.3	23.1	-	-	-
6.	Counselling plus medication to treat nicotine withdrawal is more effective than either intervention alone?	69.0	12.0	19.0	-	-	-
7.	A common withdrawal symptom that occurs after quitting smoking is weight loss.	28.2	46.8	25.0	-	-	-
8.	Smokers who are highly nicotine dependent, have social stressors and psychiatric comorbidities, are less likely to be successful at quitting?	71.1	15.4	13.5	-	-	-
9.	Patients who have their first cigarette within half an hour of waking are likely to be less dependent on nicotine than patients who have it much later in the day.	10.9	48.1	41.0	-	-	-
10.	Most smokers will successfully quit smoking on their own without assistance.	37.2	54.5	8.3	-	-	-
11.	When advising patients to stop smoking, the advice should never be linked to the patient’s current health or illness.	30.8	66.7	2.5	-	-	-
12.	Counselling patients on smoking cessation includes assisting the patient to set a quit date.	60.9	31.4	7.7	-	-	-
13.	Most of the withdrawal symptoms from smoking cessation disappear within 4 weeks of abstinence.	31.4	19.9	48.7	-	-	-
14.	It is imperative to speak to adolescents in the presence of their caregiver when encouraging smoking cessation.	55.8	34.0	10.2	-	-	-
15.	There is no need of advising elderly patients who smoke (those above 60 years) to quit as the damage from smoking is already present and cannot be reversed.	11.0	86.0	3.0	-	-	-
16.	Smokers have double the risk of developing TB and of dying of TB than non-smokers.	80.8	15.4	3.8	-	-	-
17.	Smoking during pregnancy is safe for the baby.	1.3	96.8	1.9	-	-	-
18.	There is no drug–drug interaction between antiretroviral drugs and medicines used for smoking cessation.	17.9	35.9	46.2	-	-	-
19.	Which of the following medications are recommended for the treatment of tobacco dependence in SA?						
	Nicotine Patch	-	-	-	73.7	5.1	21.2
	Nicotine gum	-	-	-	72.4	6.4	21.2
	Nicotine lozenge	-	-	-	51.9	6.4	41.7
	Nicotine syrup	-	-	-	11.0	86.0	3.0
	Bupropion	-	-	-	15.4	11.5	73.1
	Varenicline	-	-	-	12.8	9.6	77.6
	Clonidine	-	-	-	6.0	17.0	77.0
	Electronic cigarettes	-	-	-	30.1	17.3	52.6
	Nortryptyline	-	-	-	15.0	14.0	71.0

TB, Tuberculosis.

### Assessment of respondents’ knowledge about smoking cessation

The majority of respondents (*n* = 119), 76.3% had poor knowledge (< 60%), 21.2% (*n* = 33) had moderate knowledge (60% – 79%), while 2.6% (*n* = 4) had good knowledge (80% – 100%).

The majority of the respondents answered correctly to the following knowledge items ‘Patients should only be asked about their smoking history if they have a smoking-related disease or illness’, ‘Smoking cessation is not a critical component of management of Chronic Obstructive Pulmonary Disease’, ‘It is not necessary to assess nicotine dependence prior to initiation of Nicotine Replacement Therapy’, ‘When advising patients to stop smoking, the advice should never be linked to the patient’s current health/illness’. In others, the majority answered either incorrectly or were not sure of the following knowledge items: ‘Patients who have their first cigarette within half an hour of waking are likely to be less dependent on nicotine than patients who have it much later in the day’, ‘Most of the withdrawal symptoms from smoking cessation disappear within 4 weeks of abstinence’. See Table 2 for other knowledge items.

Table 3 shows the respondents’ attitude scores. Most respondents strongly agree that smoking cessation counselling is an important part of their job.

**TABLE 3:** Respondents’ attitude score.

No	Attitude item	Strongly agree (%)	Agree (%)	Neutral (%)	Disagree (%)	Strongly disagree (%)
1	Smoking cessation counselling is an important part of my job.	72.4	17.3	5.8	2.6	1.9
2	It’s not worth discussing benefits of smoking cessation with patients as patients already know they should quit.	18.0	3.0	7.0	22.0	50.0
3	Smoking is a personal decision which does not concern the health care worker.	9.6	10.9	4.5	21.8	53.2
4	My patients’ acute health problems take precedence over smoking counselling.	12.8	27.6	25.6	16.7	17.3
5	Patients are not receptive to receiving smoking cessation assistance from healthcare providers.	15.4	28.8	25.6	20.5	9.6
6	Smoking cessation counselling negatively affects my relationship with patients.	5.8	8.3	9.6	44.9	31.4
7	Clinicians should discuss smoking cessation with patients even if it’s not the reason for the visit.	52.0	42.0	6.0	0.0	0.0
8	I do not have sufficient time to provide advice and counselling to all my patients who smoke during routine consultations.	12.2	26.3	11.5	32.1	17.9
9	It is uncomfortable to counsel my smoking patients on quitting smoking.	6.4	7.1	9.6	38.5	38.5
10	Patients do not comply with information given on smoking cessation.	19.0	45.0	20.0	11.0	5.0
11	Patients are not interested in receiving smoking cessation information.	10.9	26.3	21.8	34.6	6.4
12	Smoking status should be included as one of the vital signs for patients attending primary care facilities.	51.3	33.3	9.0	3.8	2.6

Most of the respondents disagree with the items ‘Smoking is a personal decision which does not concern the health care worker’ (*n* = 117), 75%, ‘It is uncomfortable to counsel my smoking patients on quitting smoking’ (*n* = 120), 77%, and ‘Patients are not interested in receiving smoking cessation information’ (*n* = 64), 41%. However, many agree with the item, ‘Clinicians should discuss smoking cessation with patients even if it’s not the reason for the visit’ (*n* = 147) 94%. See Table 3 for respondents’ agreement with the other attitude items.

Table 4 depicts the respondents’ practice scores, with the majority of respondents sometimes asking patients about their smoking status. Still, many sometimes or never record the patients smoking history in their medical records.

**TABLE 4:** Respondents’ practice score.

No	Practice item In the last month how frequently did you do the following:	Never (%)	Sometimes (%)	Always (%)
1	Ask patients about their smoking status?	5.8	51.3	42.9
2	Ask the patients on how many cigarettes they smoke a day?	9.6	51.9	38.5
3	Record the patients’ smoking history in their medical records.	15.4	37.8	46.8
4	Discuss the risks of smoking and benefits of quitting smoking with patients?	5.1	44.9	50.0
5	Advise patients to quit smoking?	3.0	31.0	66.0
6	Ask patients about previous attempts to quit smoking?	19.9	53.2	26.9
7	Assess a smoking patient on willingness to quit smoking?	18.6	48.1	33.3
8	Encourage the use of nicotine replacement therapy?	38.5	43.6	17.9
9	Assist a smoking patient to set up a target quit date?	40.3	38.5	21.2
10	Arrange follow up visits to discuss quitting?	54.5	30.1	15.4
11	Encourage not to smoke in the presence of infants and children.	9.0	17.3	73.7

Many respondents sometimes encourage the use of NRT (*n* = 68), 43.6% and always encourage patients not to smoke in the presence of infants and children (*n* = 115), 73.7%. See Table 4 for healthcare providers’ responses for other practice items.

Table 5 shows the respondents’ perceived barriers to smoke cessation counselling. The top three perceived barriers to smoking cessation counselling, in descending order, were ‘Lack of pharmaceutical medication for Nicotine Replacement Therapy’ (*n* = 109), 70%, ‘Lack of copies of smoking cessation guidelines in the facility’ (*n* = 100), 64.1%, and ‘Some health care providers are themselves smokers’ (*n* = 95), 60.9%.

**TABLE 5:** Perceived barriers to smoking cessation counselling.

No	Barrier	Not a barrier	Somewhat a barrier	Important barrier
1	A lack of time	24.4	42.9	32.7
2	A lack of community organisations to refer patients to	10.9	29.5	59.6
3	A lack of patient educational material (brochures or pamphlets)	16.6	37.2	46.2
4	A lack of knowledge and training on smoking cessation counselling	12.8	42.3	48.9
5	A lack of copies of smoking cessation guidelines in the facility	9.0	26.9	64.1
6	A lack of pharmaceutical medication for nicotine replacement therapy	9.0	21.0	70.0
7	Patients have more immediate health problems to be addressed	27.0	40.0	33.0
8	Some healthcare providers are themselves smokers	11.5	27.6	60.9
9	Quitting smoking is stressful to patients	12.8	46.2	41.0

Table 6 shows an analysis of the background characteristics of respondents with KAP. It indicates that the healthcare practitioners’ smoking status was significantly associated with the practice of assessing a smoking patient’s willingness to quit smoking (*p* = 0.033). When knowledge was compared with some variables, knowledge was significantly associated with profession (*p* ≤ 0.001) and year of practice (*p* = 0.008) but was not associated with smoking status (*p* = 0.479).

**TABLE 6:** Bivariate analysis of background characteristics versus knowledge, attitude and practice.

Demographic characteristics														
KAP items	Rating	Smoking status			*p*	Profession or rank				*p*	Years of practice			*p*
		Never smoked (%)	Ex-smoker (%)	Current smoker (%)		Medical officer (%)	Professional nurse (%)	Enrolled nurse (%)	Enrolled nursing assistant (%)		0–5 (%)	6–10 (%)	> 10 (%)	
**Knowledge**	Good	3.2	0.0	0.0	0.479	11.1	2.1	0.0	0.0	**< 0.001**	3.6	0.0	3.3	**0.008**
	Moderate	21.6	31.2	6.7	-	55.6	20.2	0.0	12.5		39.3	28.9	12.3	
	Poor	75.2	68.8	93.3	-	33.3	77.7	100.0	87.5		57.1	71.1	84.4	
**Attitude**	Strongly agree	81.4	8.9	9.7	0.274	8.9	61.9	8.9	20.4	0.284	15.0	23.9	61.1	0.506
Smoking cessation counselling is an important part of my job.	Agree	81.5	7.4	11.1	-	22.2	59.3	3.7	14.8	-	22.2	25.9	51.9	-
	Neutral	66.7	33.3	0.0	-	11.1	33.3	11.1	44.4	-	22.2	33.3	44.4	-
	Disagree	50.0	25.0	25.0	-	0.0	100.0	0.0	0.0	-	25.0	25.0	50.0	-
	Strongly disagree	100.0	0.0	0.0	-	33.3	33.3	0.0	33.3	-	66.7	0.0	33.3	-
My patients’ acute health problems take precedence over smoking counselling	Strongly agree	75.0	10.0	15.0	0.075	25.0	45.0	10.0	20.0	**0.002**	20.0	20.0	60.0	0.118
	Agree	79.1	13.9	7.0	-	13.9	53.5	4.7	27.9	-	9.3	32.6	58.1	-
	Neutral	77.5	15.0	7.5	-	7.5	47.5	17.5	27.5	-	27.5	17.5	55.0	-
	Disagree	76.9	0.0	23.1	-	7.7	69.2	3.9	19.2	-	7.7	38.5	53.9	-
	Strongly disagree	92.6	7.4	0.0	-	7.4	92.6	0.0	0.0	-	25.9	11.1	63.0	-
Smoking cessation counselling negatively affects my relationship with patients	Strongly agree	77.8	11.1	11.1	0.114	11.1	33.3	0.0	55.6	**0.001**	11.1	11.1	77.8	0.095
	Agree	84.6	0.0	15.4	-	0.0	53.9	7.7	38.5	-	30.8	38.5	30.8	-
	Neutral	86.7	0.0	13.3	-	20.0	26.7	33.3	20.0	-	0.0	33.3	66.7	-
	Disagree	75.7	18.6	5.7	-	15.7	60.0	5.7	18.6	-	17.1	28.6	54.3	-
	Strongly disagree	83.7	4.1	12.2	-	6.1	77.6	4.1	12.2	-	22.5	14.3	63.3	-
Clinicians should discuss smoking cessation with patients even if it’s not the reason for the visit.	Strongly agree	87.7	7.4	4.9	**0.008**	9.9	64.2	7.4	18.5	0.882	13.6	24.7	61.7	0.118
	Agree	76.9	10.8	12.3	-	13.9	56.9	7.7	21.5	-	23.1	20.0	56.9	-
	Neutral	33.3	33.3	33.3	-	11.1	44.4	11.1	33.3	-	22.2	55.6	22.2	-
	Disagree	0.0	0.0	0.0	-	0.0	0.0	0.0	0.0	-	0.0	0.0	0.0	-
	Strongly disagree	100.0	0.0	0.0	-	0.0	100.0	0.0	0.0	-	0.0	0.0	100.0	-
I do not have sufficient time to provide advice and counselling to all my patients who smoke during routine consultations	Strongly agree	84.2	15.8	0.0	0.327	21.1	68.4	5.3	5.3	0.130	15.8	0.0	84.2	**< 0.001**
	Agree	80.5	12.2	7.3	-	19.5	63.4	7.3	9.8	-	39.0	26.8	34.2	-
	Neutral	72.2	11.1	16.7	-	5.6	50.0	5.6	38.9	-	16.7	38.9	44.4	-
	Disagree	78.0	12.0	10.0	-	6.0	64.0	8.0	22.0	-	8.0	26.0	66.0	-
	Strongly disagree	85.7	0.0	14.3	-	7.1	50.0	10.7	32.1	-	7.1	25.0	67.9	-
**Practice**														
Ask the patients on how many cigarettes they smoke a day?	Never	60.0	20.0	20.0	**< 0.001**	6.7	66.7	20.0	6.7	**0.049**	33.3	0.0	66.7	0.100
	Sometimes	77.8	12.4	9.9	-	6.2	64.2	4.9	24.7	-	16.1	28.4	55.6	-
	Always	88.3	5.0	6.7	-	20.0	53.3	8.3	18.3	-	16.7	25.0	58.3	-
Record the patients smoking history in their medical records	Never	62.5	16.7	20.8	0.055	0.0	41.7	25.0	33.3	**< 0.001**	12.5	20.8	66.7	0.920
	Sometimes	81.3	6.8	11.9	-	5.1	72.9	3.4	18.6	-	18.6	23.7	57.6	-
	Always	84.9	11.0	4.1	-	20.6	56.2	5.5	17.8	-	19.2	26.0	54.8	-
Discuss the risks of smoking and benefits of quitting smoking with patients?	Never	57.1	28.6	14.3	0.294	0.0	28.6	71.4	0.0	**< 0.001**	4.3	0.0	85.7	0.123
	Sometimes	80.3	12.7	7.0	-	15.5	62.0	2.8	19.7	-	22.5	21.1	56.3	-
	Always	81.8	6.5	11.7	-	9.1	62.3	6.5	22.1	-	13.0	29.9	57.1	-
Ask patients about previous attempts to quit smoking?	Never	64.5	19.4	16.1	**0.044**	12.9	61.3	9.7	16.1	0.933	32.3	12.9	54.8	0.154
	Sometimes	83.1	10.9	6.0	-	12.1	60.2	8.4	19.3	-	13.3	28.9	57.8	-
	Always	85.7	2.4	11.9	-	9.5	59.5	4.8	26.2	-	16.7	23.8	59.5	-
Assess a smoking patient on willingness to quit smoking?	Never	69.0	10.3	20.7	**0.033**	3.5	75.9	3.5	17.2	**0.017**	31.0	13.8	55.2	0.059
	Sometimes	85.3	12.0	2.7	-	17.3	54.7	13.3	14.7	-	16.0	20.0	64.0	-
	Always	78.9	7.7	13.5	-	7.7	59.6	1.9	30.8	-	13.5	36.5	50.0	-

Note:Bold values, Statistically significant difference.

KAP, knowledge, attitude and practice.

There was a significant association (*p* = 0.002 and *p* = 0.001) between the professional ranking and their responses to the items ‘My patients’ acute health problems take precedence over smoking counselling’ and ‘Smoking cessation counselling negatively affects my relationship with patients’, respectively. An association was found between the item ‘Clinicians should discuss smoking cessation with patients even if it’s not the reason for the visit’ and the smoking status of the HCPs (*p* = 0.008). ‘I do not have sufficient time to provide advice and counselling to all my patients who smoke during routine consultations’ was significantly associated with HCPs’ years of practice (*p* ≤ 0.001).

Asking the patients how many cigarettes they smoke a day was significantly associated with HCP’s smoking status (*p* ≤ 0.001) and profession (*p* = 0.049) but not their years of practice (*p* = 0.100). A significant association was found for items ‘recording the patient’s smoking history in their medical records’, ‘discussing the risks of smoking and benefits of quitting smoking’ with HCPs profession (*p* ≤ 0.001). Asking patients about previous attempts to quit smoking was significantly associated with the smoking status of the HCPs (*p* = 0.044). Assessing a smoking patient on willingness to quit smoking was also significantly associated with HCP’s smoking status (*p* = 0.033) and profession (*p* = 0.017).

Table 7 compares the association between KAP items.

**TABLE 7:** Associations between knowledge, attitude and practice.

Attitude		Knowledge			*P*
Items	Scale	Good (%)	Moderate (%)	Poor (%)	
Smoking cessation counselling is an important part of my job.	Strongly agree	1.8	20.4	77.9	0.332
	Agree	3.7	22.2	74.1	-
	Neutral	0.0	33.3	66.7	-
	Disagree	0.0	25.0	75.0	-
	Strongly disagree	33.3	0.0	66.7	-
My patients’ acute health problems take precedence over smoking counselling.	Strongly agree	15.0	20.0	65.0	0.214
	Agree	0.0	20.9	79.1	-
	Neutral	0.0	20.0	80.0	-
	Disagree	3.85	19.23	76.9	-
	Strongly disagree	0.0	25.9	74.1	-
Smoking cessation counselling negatively affects my relationship with patients.	Strongly agree	22.2	33.3	44.4	**< 0.001**
	Agree	0.0	0.0	100.0	-
	Neutral	6.7	13.3	80.0	-
	Disagree	1.4	32.9	65.7	-
	Strongly disagree	0.0	10.2	89.8	-
Clinicians should discuss smoking cessation with patients even if it’s not the reason for the visit.	Strongly agree	2.5	22.2	75.3	0.826
	Agree	3.1	18.5	78.5	-
	Neutral	0.0	33.3	66.7	-
	Disagree	0.0	0.0	0.0	-
	Strongly disagree	0.0	0.0	100.0	-
I do not have sufficient time to provide advice and counselling to all my patients who smoke during routine consultations.	Strongly agree	15.8	21.1	63.2	0.051
	Agree	2.4	29.3	68.3	-
	Neutral	0.0	27.8	72.2	-
	Disagree	0.0	18.0	82.0	-
	Strongly disagree	0.0	10.7	89.3	-
**Practice**					
Ask the patients on how many cigarettes they smoke a day?	Never	6.7	13.3	80.0	**0.049**
	Sometimes	0.0	17.3	82.7	-
	Always	5.0	28.3	66.7	-
Record the patients smoking history in their medical records	Never	0.0	12.5	87.5	0.571
	Sometimes	3.4	18.6	78.0	-
	Always	2.7	26.0	71.2	-
Discuss the risks of smoking and benefits of quitting smoking with patients?	Never	0.0	14.3	85.7	0.494
	Sometimes	2.8	28.2	69.0	-
	Always	2.6	15.6	81.8	-
Ask patients about previous attempts to quit smoking?	Never	6.5	22.6	71.0	**0.023**
	Sometimes	0.0	15.7	84.3	-
	Always	4.8	31.0	64.2	-
Assess a smoking patient on willingness to quit smoking?	Never	3.5	24.1	72.4	0.823
	Sometimes	1.3	21.3	77.3	-
	Always	3.9	19.2	76.9	-

Bold values, Statistically significant difference.

Knowledge was not significantly associated with the item, ‘clinicians should discuss smoking cessation with patients even if it’s not the reason for the visit’. When attitude items were compared with knowledge, no significance was found except for the item ‘Smoking cessation counselling negatively affects my relationship with patients’ (*p* ≤ 0.001). Practice items, such as asking the patients how many cigarettes they smoke daily and previous attempts to quit smoking, were strongly associated with knowledge (*p* = 0.049 and 0.023), respectively.

## Discussion

Smoking cessation counselling and the pharmacological treatment HCPs deliver are crucial in helping smokers quit. This study investigated primary HCPs’ KAP on smoking cessation interventions in the Northern Cape, Sol Plaatje district.

### Knowledge

This study included several HCPs with various levels of knowledge about smoking cessation guidelines and has identified several issues. Among the findings, most HCPs lacked knowledge of existing smoking cessation guidelines. This is similar to findings in a study by Mahoto, Mitonga and Oladimeji (2023), where most HCPs in Zambezi region, Namibia, were unaware of the national tobacco legislation. Batini et al. (2019) reported that HCPs are poorly equipped to support smokers willing to quit smoking. Banandur, Kumar and Gopalkrishna (2017) reported that in India, most of the persons responsible for compliance and authorised officers lacked appropriate awareness of the provisions in the Cigarette and Other Tobacco Products Act (COPTA) guidelines. The reasons for the lack of knowledge of the existing guidelines, as found by our study, may be because of the lack of priority on the subject; there appears to be no any form of continuing HCP education on smoking cessation in the study setting.

Most HCPs knew that smoking cessation advice given by a healthcare professional increased the patient’s chances of quitting smoking. This was similar to findings by He et al. (2021) in a study from China, which reported that doctors’ advice was associated with a smoker’s intention and action to quit smoking. Most of the HCPs were knowledgeable that most smokers would not successfully quit smoking without assistance. This was supported by similar suggestions from other studies on smoking cessation (Batini et al. 2019; He et al. 2021; Tamirat 2021; Van Zyl-Smit et al. 2013). Thus, despite HCPs’ awareness of the positive effects of smoking cessation counselling, there seems to be a lack of motivation to practice it.

Many HCPs were knowledgeable that smoking increases the risk of developing tuberculosis and mortality from tuberculosis, as reported in other studies (Batini et al. 2019; Van Zyl-Smit et al. 2013). Almost all HCPs were knowledgeable that smoking during pregnancy is unsafe for the baby. In a study involving midwives in the public sector antenatal clinics, Murphy, Steyn and Matthews (2016) reported that all midwives agreed that smoking during pregnancy harms the unborn baby. Chamberlain et al. (2017), in their study, reported that psychosocial interventions to support pregnant women could increase the number of women who stop smoking in late pregnancy and decrease the number of babies born with low birth weight.

Despite the existence of pharmacological interventions for smoking cessation and possible drug–drug interaction with other medications the patient might be taking, the HCPs in this study were more knowledgeable of only one type of pharmacological intervention – NRT. They were not knowledgeable about the other pharmacological modalities such as antidepressants and nicotine receptor agonists (NRA). The HCPs knew about NRT probably because it is the most commonly known of the pharmacological interventions but was not readily available in our study setting. Similar to this finding, a systematic review by Ilesanmi, Agwai and Afolabi (2021) reported a low percentage of physicians who prescribed pharmaceutical interventions to help patients quit smoking. This was because of poor knowledge and the unavailability of NRT.

Most HCPs were knowledgeable that combining counselling and medication was more effective than either intervention alone, which was confirmed by other studies reviewed (Banandur et al. 2017; Ilesanmi et al. 2021; Van Zyl-Smit et al. 2013). The combination of pharmacological medications and counselling will assist in addressing the psychosocial influence that causes a person to smoke or prevent them from overcoming their smoking addictions.

When overall knowledge was categorised in this study, most of the HCPs were found to have poor knowledge about smoking cessation. This is in keeping with findings from other studies on smoking cessation (Ilesanmi et al. 2021; Mahoto et al. 2023). The lack of training of HCPs on smoking cessation methods and non-implementation of available guidelines are likely to be blamed for the poor knowledge in our study setting.

### Attitude

Generally, smoking often leads to several adverse health consequences and is considered a basic health education topic that HCPs should emphasise. This study found that most HCPs agreed that counselling on smoking was an important part of their job. This was supported by other studies (De Frel et al. 2022; Ilesanmi et al. 2021; Van Zyl-Smit et al. 2013). However, Mahoto et al. in their study in the Zambezi region, reported that although the majority of HCPs regard themselves as providers of preventive healthcare, they do not necessarily consider the provision of smoking cessation intervention as part of their duty (Mahoto et al. 2023).

Offering basic smoking cessation advice is likely to assist in further encouraging those contemplating quitting to take the bold step. Most HCPs in this study disagreed with the statement that it is not worth discussing the benefits of smoking cessation with patients as they already know they should quit. Carsten and Linley share a similar view, as they suggested that even with minimal advice, the likelihood of patients quitting smoking increases (Carstens & Linley 2020).

Healthcare providers were unsure if the patients’ acute health conditions should take precedence over smoking counselling. Their uncertainty regarding this statement is understandable; while the significance of smoking cessation is undisputable, it is crucial to consider the timing of this intervention when faced with acutely ill patients. This is in keeping with findings by Mahoto et al. (2023) who reported that addressing the patients’ immediate healthcare needs took precedence over smoking cessation counselling.

Half of the HCPs disagree that time constraints are an issue for them. This contrasts with reports from other studies where time constraints have been noticed to be a reason why HCPs skip smoking cessation counselling (De Frel et al. 2022; Mahoto et al. 2023). Despite time constraints in healthcare delivery, incorporating vital health promotion counselling remains crucial and can be achieved through effective time management.

The majority of HCPs agree that patients do not comply with the information given on smoking cessation, and this was supported by a report by Klink et al. (2011) in a study involving HCPs in China, where only 13.3% of the respondents believe their patients will comply. Although patients have to take responsibility for their health, designing healthcare interventions to enhance compliance with HCPs’ advice should be encouraged. Most HCPs also agree that smoking should be included as a ‘vital sign’ for patients attending primary care facilities in keeping with the guidelines (Van Zyl-Smit et al. 2013). This will assist in reminding HCPs to provide smoking cessation counselling during the consultation with patients.

### Practice: Using the 5A model of brief intervention

#### Ask

In their daily practice, it is recommended that HCPs ask for and record the smoking status of their patients (Garies et al. 2020; Van Schayck et al. 2017; Van Zyl-Smit et al. 2013). This may also help to monitor the impact of smoking cessation interventions (Garies et al. 2020). This study found poor and inconsistent record-keeping; less than half of the HCPs always ask for and record their patients’ smoking status. This may be because of the omission of smoking status in the history-taking or forgetfulness in documentation despite asking. This is similar to findings in Ethiopia, where only 28% of healthcare workers asked about patients’ smoking status and 21.3% kept a record of this status (Tamirat 2021), but contrasts with a survey in Finland, where smoking status was documented in 60% of patients with chronic diseases and clinicians discussed smoking cessation with 49% of patients who were current smokers (Fitzpatrick et al. 2023).

Less than half of the HCPs always asked their patients how many cigarettes they smoked a day. This information is necessary to determine the level of nicotine dependency and the intervention required (Van Schayck et al. 2017). Papadakis et al. (2020) reported in their study in Greece that respondents reported smoking 24.6 cigarettes per day for a mean duration of 32.4 years. Van Schayck et al. (2017) suggested that pharmacotherapy should be offered to those smoking more than 10 cigarettes daily.

#### Advise and assess willingness to quit smoking

Routinely advising patients on the risks associated with smoking during the consultation may increase the number of those who successfully quit smoking. Above half of the HCPs in this study always advised their patients on the benefits of smoking cessation and offered advice to quit smoking. Similar advice was reported to have been given to patients in other smoking cessation studies (Fitzpatrick et al. 2023; Papadakis et al. 2020; Tamirat 2021). Tamirat (2021), in a study in Ethiopia, said that only 24.2% of HCPs surveyed always advised patients to quit smoking. Tamirat (2021) also reported that only 18.8% of HCPs always asked about previous attempts to quit smoking, and 19.4% always assessed their willingness to quit smoking at that time. This is comparable to findings in our study, where only about a third of the HCPs asked similar questions. Healthcare providers understanding a patient’s smoking history, previous attempts and willingness to quit smoking will ensure that they come up with a smoking cessation plan that is more likely to succeed and avoid triggers that may cause the patient to relapse.

#### Assist and arrange follow-up

The International Primary Care Respiratory Group (IPCRG) and the South African Tobacco Smoking Cessation Clinical Practice Guidelines, as part of the 5A model, recommend assisting a smoker in setting up target quit dates and arranging follow-up visits (Van Schayck et al. 2017; Van Zyl-Smit et al. 2013). In this study, less than a third of HCPs always complied with the guidelines, comparable to findings by Tamirat where 9.2%, 9.6% and 8.6% HCPs assisted patients in setting up target quit dates, arranged follow-up visits, and encouraged the use of NRT respectively (Tamirat 2021). Healthcare providers, by assisting patients in setting up a target quit date, ensure they have enough time to prepare psychologically and eliminate items such as cigarettes and ashtrays in their environments. Start hobbies that can assist them to forget about smoking and seek support groups or family support to motivate them further.

#### Barriers

This study has also revealed some of the barriers to the provision of smoking cessation. Almost half of the HCPs described a lack of knowledge and training on smoking cessation counselling as an important barrier. This is conversant with reports from other studies where a lack of knowledge was identified as a barrier (Ilesanmi et al. 2021; Klink et al. 2011; Mahoto et al. 2023; Tamirat 2021). Healthcare providers who receive training are more likely to provide smoking cessation interventions (Tamirat 2021).

Most HCPs also reported the lack of copies of guidelines in the facilities as a barrier. In a systemic review by Ilesanmi et al. (2021), they said that in one of the studies reviewed, only 17% of HCPs had guidelines to help patients quit smoking (Ilesanmi et al. 2021). This is likely to negatively impact the knowledge of new health workers who are unlikely to have come across the guidelines.

Most HCPs reported a lack of community organisations to refer the patients to as an important barrier to smoking cessation counselling. A similar situation was also reported by other studies reviewed (Ilesanmi et al. 2021; Mahoto et al. 2023). This may be because community support organisations offer patients the opportunity to meet with other smokers with a similar experience and may provide individualised support. Pamphlets and brochures assist HCPs in starting an educational conversation with a smoker and provide support and motivation for those trying to quit. Almost half of the HCPs in this study described a lack of these educational materials as an important barrier, as other studies also found (Ilesanmi et al. 2021; Mahoto et al. 2023).

Most HCPs believe that smoking by HCPs can act as an important barrier to smoking cessation counselling. In a systematic review and meta-analysis of the prevalence of tobacco use among HCPs, Nilan et al. (2019) reported a total prevalence of 19% among all HCPs. This is a cause of concern because these are the same people tasked with encouraging smoking cessation in the general population and are required to be role models (Van Schayck et al. 2017). Smoking cessation guidelines recommend that all HCPs provide smoking cessation advice to smoking patients. Healthcare providers who smoke are unlikely to follow this recommendation (Duaso et al. 2017). Even if they do, their habits will likely undermine whatever they say. Regardless of their smoking status, they should be encouraged to provide this service to all smoking patients (Nilan et al. 2019). There is a need to offer smoking cessation interventions as a priority to all smoking HCPs (Evenhuis et al. 2023).

## Conclusion

This study found that most HCPs from Sol Plaatje Municipality within the Northern Cape need to acquire knowledge of existing smoking cessation guidelines. However, most HCPs within this municipality know that smoking cessation advice from a healthcare professional increases the chances of patients quitting smoking and that most smokers would only successfully quit with assistance. Many HCPs also knew about the increased risk of tuberculosis due to smoking.

These findings suggest that HCPs need increased awareness and education about smoking cessation guidelines to improve care for individuals who smoke or use tobacco products.

### Limitations and strengths of the study

The study benefitted from a high response rate from the required number of respondents, which enhanced the accuracy and data quality. However, as the study was conducted in only one municipality in the Northern Cape, the external validity of applying these findings to the entire Northern Cape and South Africa is limited. Furthermore, the use of self-reported interviews with HCPs may have introduced bias, as they might have over-reported their provision of smoking cessation services. Additionally, the study’s ability to differentiate between the expected knowledge level for each professional group was limited, as a doctor may be more likely to possess better smoking cessation guideline knowledge compared with a professional nurse.
